# Immunomodulatory Tissue Factors in the Gallbladder Walls of Pediatric Patients with Chronic Calculous Cholecystitis

**DOI:** 10.3390/children12020205

**Published:** 2025-02-08

**Authors:** Kaiva Zīle Zariņa, Māra Pilmane, Aigars Pētersons

**Affiliations:** 1 Institute of Anatomy and Anthropology, Riga Stradins University, Kronvalda Boulevard 9, LV-1010 Riga, Latvia; 2 Department of Pediatric Surgery, Riga Stradins University, Dzirciema Street 16, LV-1007 Riga, Latvia

**Keywords:** IL-12, IL-13, IL-1β, SHH, NFkBp65, HSP60, cholecystitis, immunomodulation

## Abstract

Background: The rising rates of gallstones and cholecystectomy in pediatric populations underscore the increasing concern regarding chronic cholecystitis. However, the morphopathogenesis of pediatric calculous cholecystitis is still not well understood. This study aimed to determine the expression and distribution of immunomodulatory factors interleukin-12 (IL-12), interleukin-13 (IL-13), interleukin-1β (IL-1β), sonic hedgehog protein (SHH), nuclear factor NF-kappa-B p65 subunit (NFkBp65), and heat shock protein 60 (HSP60) in the gallbladder walls of pediatric patients with chronic calculous cholecystitis. Methods: In total, 11 gallbladder samples were collected from pediatric patients with calculous cholecystitis during cholecystectomy, while 5 healthy gallbladder samples served as controls. IL-12, IL-13, IL-1β, SHH, NFkBp65, and HSP60 were detected by immunohistochemistry. The number of positive structures in gallbladder wall epithelium, vasculature, and inflammatory infiltrate was assessed semi-quantitatively by microscopy. A Mann–Whitney U test and Spearman’s rank-order correlation coefficient were calculated. Results: Statistically significant differences were observed between patient and control samples in the expression of IL-1β, SHH, and NFkBp65 in the epithelium, as well as in the expression of IL-12, SHH, and HSP60 in the blood vessels. The expression of IL-1β was stronger in the epithelium of controls, while other markers were more prominent in patient samples. Conclusions: An increased number of NFkBp65, IL-12, and HSP60 positive cells in patient gallbladder tissue suggests a significant role of these tissue factors in driving immune modulation and sustaining the inflammation in pediatric chronic calculous cholecystitis. The noticeable expression of SHH in patient gallbladder tissue indicates its part in tissue regeneration and repair processes, as well as in modulating inflammation and vascular responses in calculous cholecystitis. The significant positive correlations between the factors studied highlight the importance of their coordinated interaction and intricate crosstalk in the morphopathogenesis of calculous cholecystitis.

## 1. Introduction

Chronic cholecystitis is defined as prolonged gallbladder inflammation and can be further classified as calculous or acalculous; however, it is most commonly associated with gallstones [1]. Chronic cholecystitis is the most common inflammatory disease of the gallbladder in pediatric patients, and the majority of the cases of pediatric chronic cholecystitis are reported in females [2], with the mean age being 14 years [3]. Currently, cholelithiasis affects from 1.9% to 4% of the pediatric population [4]. There has been a consistent increase in the incidence of gallstones requiring cholecystectomy [5], yet little is known about the morphopathogenesis of pediatric calculous cholecystitis. Although gallstones in children are typically associated with hemolytic disease and biliary dyskinesia, the number of non-hemolytic cholelithiasis cases is rising. Currently, most cases of pediatric cholelithiasis are linked to obesity [6]. Despite recognizing these processes, the specific pathways and mechanisms leading to pediatric chronic calculous cholecystitis are not fully understood. Further research is required to gain a deeper understanding of the role of genetic factors, environmental influences, and immune responses in the development and progression of this disease in children. Here, we focused on the factors, cytokines, and cellular activity regulators that are known to modulate the local immune response.

Interleukin 12 (IL-12) is a major immunoregulatory cytokine produced predominantly by antigen-presenting cells. IL-12 expressing cells include dendritic cells, macrophages, monocytes, neutrophils, B cells, and microglia cells [7,8,9,10]. This cytokine is also generated by non-immune cells such as epitheliocytesl, endotheliocytes, infected keratinocytes, and osteoblasts [11]. Its immunoregulatory action is carried out by activating natural killer (NK) cells, enhancing CD8+ T cell cytolytic activity, and inducing the differentiation of naive CD4+ T lymphocytes into T helper 1 (TH1) cells. As a result, interferon-gamma (IFN-γ) is produced, and cellular immunity is promoted (Table 1). In addition, IL-12 exhibits anti-angiogenic and anti-tumor effects [12,13]. Interestingly, higher levels of circulating IL-12 have been associated with gallstone disease in adults [14]. While the importance of IL-12 as a connector between innate and adaptive immunity is undoubted during infection [15], it is not fully clear whether IL-12 is involved in the morphopathogenesis of pediatric chronic inflammatory conditions, such as calculous cholecystitis.

Interleukin-13 (IL-13) is a cytokine mainly associated with helminth infections [16] and allergic inflammation, including asthma and allergic rhinitis [17]. IL-13 is typically considered a product of T helper 2 (TH2) cells, although NK cells, CD8+ cells, B lymphocytes, mast cells, macrophages, basophils, and eosinophils partake in the expression of IL-13 as well. IL-13 shares functional resemblance with another TH2 cytokine, interleukin-4 (IL-4), with about a quarter of their amino acid sequences overlapping. Both IL-13 and IL-4 can act through a common receptor—the type II receptor, which is a heterodimer formed from an IL-4 receptor (IL-4R) α and an IL-13 receptor (IL-13R) α1 subunit [18]. Interestingly, TH1 and TH17 cells can participate in its secretion [19], while IL-13 antagonizes TH17 and TH1-mediated inflammation [20,21]. Immunological functions of IL-13 include promoting proliferation and differentiation of B lymphocytes, thus leading to the synthesis of antibodies and, importantly, participating in the immunoglobulin (Ig) class switch to IgE [22,23]. In addition, IL-13 promotes chemokine secretion, which leads to eosinophil trafficking [24]. IL-13 also has a major role in the pathogenesis of fibrotic processes [25], (Table 1). It is not yet understood if the TH2 immune response could have a role in regulating inflammation in cases of calculous cholecystitis.

Interleukin-1β (IL-1β), a member of the IL-1 family, is a proinflammatory cytokine mainly secreted by macrophages and monocytes [26]. Overall, the function of IL-1β is to mediate proinflammatory reactions as a consequence of pathogen-associated molecular patterns (PAMPs), for example, bacterial or viral products, and danger-associated molecular patterns, which are released from damaged cells (DAMPs), for example, uric acid crystals or adenosine 5′-triphosphate [27]. IL-1β is a key element needed for T helper 17 (TH17) cell differentiation [28], (Table 1). It should be noted that this cytokine is produced as an inactive precursor, pro-IL-1β, which is then cleaved by caspase-1 to its biologically active form. For activation of caspase-1, an inflammasome is required [29]. A key component of an inflammasome is the NLRP3 protein [30], and gain-of-function mutations in the NLRP3 gene result in higher levels of IL-1β, leading to autoinflammatory diseases [31].

Sonic hedgehog protein (SHH) is a signaling protein crucial for embryonic development, organogenesis, and cell differentiation. Loss of SHH during embryonic development leads to significant defects, affecting the central nervous system and causing holoprosencephaly, as well as impacting the skeletal system and gut and lung development [32]. In adult tissues, SHH is responsible for tissue homeostasis by regulating differentiation and proliferation and tissue repair and regeneration [33]. SHH has an important role in multiple tissues postnatally, including the central nervous system, tissues of ectodermal origin, such as the skin and teeth, as well as in tissues of mesodermal origin, for example, bone and muscle. The expression of SHH is also detected in respiratory and gastrointestinal systems [32]. In addition, the SHH pathway affects the immune system by regulating proliferation and differentiation of thymocyte progenitors and by modulating T-cell receptor signaling and T-cell differentiation [34], (Table 1). Impaired SHH signaling is found in various cancers, for instance, pancreas, prostate, breast, and brain tumors [35]. It has previously been noted that the expression of SHH is increased in gallbladders with chronic cholecystitis and gallbladder carcinoma of adult patients [36], yet the role of SHH in pediatric gallbladder disease remains poorly understood. Viewing the expression of SHH in context with different immunomodulatory factors may contribute to a better understanding of the specific pathogenetic mechanisms.

The nuclear factor NF-kappa-B p65 subunit (NFkBp65), also known as RelA protein, is one of the five members of the NF-κB transcription factor family. NFkBp65 is involved in the formation of the most abundant NF-kB heterodimer [37], and NF-kB is present in almost all cell types [38]. Two NF-κB pathways are known—the canonical and non-canonical [39]. NF-kB is essential for innate and adaptive immune responses—it drives inflammation both by boosting the production of inflammatory cytokines, including Il-12, chemokines, and adhesion molecules, and by controlling key cellular processes, namely, proliferation, apoptosis, morphogenesis, and differentiation in various immune cells (Table 1). Among the targets of NF-kB signaling are T cells, where this transcription factor modulates activation, differentiation, and effector function. Another important aspect of NF-kB actions is the regulation of inflammasomes [40]. Abnormal activation of NF-kB is linked to various inflammatory, autoimmune, and malignant diseases [41], suggesting that its expression may be deviant in cases of pediatric calculous cholecystitis.

The 60 kDa heat shock protein (HSP60), also known as chaperonin 60 (Cpn60), is a molecular chaperone that is responsible for correct protein folding [42]. Overall, HSPs are induced by conditions of stress, including elevated temperature, hypoxia, and oxidative damage [43]. Most of the intracellular HSP60 molecules are localized in mitochondria, while the remaining part can be found in the cytoplasm. Intracellular HSP60 contributes to cellular protein homeostasis by facilitating the folding of misfolded proteins and supporting proper protein folding, trafficking, assembly, and degradation [44]. On the cell surface, HSP60 exhibits actions of a danger signaling molecule, therefore stimulating the immune system [45]. Extracellular HSP60 induces cytokine production and upregulates the expression of toll-like receptors (TLR) [46], (Table 1). Interestingly, HSP60 can also act as an anti-inflammatory modulator depending on concentration—low concentration of HSP60 is associated with suppressed activity of T regulatory cells and impaired migration of effector T cells [47].

Consequently, this study aimed to determine the expression and distribution of interleukin-12 (IL-12), interleukin-13 (IL-13), interleukin-1β (IL-1β), sonic hedgehog protein (SHH), nuclear factor NF-kappa-B p65 subunit (NFkBp65), and heat shock protein 60 (HSP60) in the gallbladder walls of pediatric patients with calculous cholecystitis and in controls.

The primary endpoint of this research is the following—expression levels of immunomodulatory markers (IL-12, IL-13, IL-1β, SHH, NFkBp65, and HSP60) in gallbladder epithelium, blood vessels, and inflammatory infiltrate. The secondary endpoints are the following: correlations between marker expression levels in the presence of chronic cholecystitis and a comparison of marker expression between epithelial cells, blood vessels, and inflammatory infiltrate.

## 2. Materials and Methods

### 2.1. Patients

Eleven gallbladder samples were obtained from pediatric patients with chronic calculous cholecystitis during laparoscopic cholecystectomy, and all of the samples were included in the study. Intraoperative steps of laparoscopic cholecystectomy included port placement, gallbladder dissection, division of cystic artery and duct, gallbladder separation from the liver, and gallbladder removal. The age of patients ranged from 12 to 17 years; all patients were females. An archive material of Riga Stradins University Institute of Anatomy and Anthropology was used. Research on archive material was carried out in the years 2023–2024. The inclusion criteria were the following: a proven presence of calculous cholecystitis by ultrasonography, a remission period of chronic cholecystitis (no exacerbations), and the absence of gallbladder anomalies. The exclusion criteria were the following: exacerbation of the disease, presence of gallbladder anomalies, and intensive medical treatment. Five healthy gallbladder samples were used for controls, control patients were aged 9 to 17, with only girls included in the study. Control samples were obtained during post-mortem autopsies from children with the cause of death being mechanical injury and with no known gallbladder disease, as well as with no signs of chronic inflammation in routine histology. All experiments were conducted according to the ethical standards expressed in the 1964 Declaration of Helsinki. This study was approved by the Riga Stradins University Ethics Committee, dated 10 May 2007. Written informed consent was obtained from parents in each case.

### 2.2. Microscopy

#### 2.2.1. Routine Microscopy

Tissue samples, including the full wall of the gallbladder, were obtained from the region between the gallbladder fundus and neck, sized approximately 2-4 mm. Gallbladder samples were fixed in a solution of 2% formaldehyde and 0.2% picric acid in 0.1 M phosphate buffer with a pH of 7.2. Furthermore, the tissues were rinsed in Tyrode’s solution containing 10% sucrose for 12 h. Following this, the samples were embedded in paraffin and sectioned into thin slices measuring 6–7 micrometers (µm). Standard histological staining with hematoxylin and eosin was applied to all samples. Each specimen was subsequently examined under bright-field microscopy to assess the morphological structure of the gallbladder wall in patients and controls.

#### 2.2.2. Immunohistochemistry

Markers IL-12, IL-13, IL-1β, SHH, NFkBp65, and HSP60 were detected by immunohistochemistry in 11 samples of patients and in 5 control samples. The biotin-streptavidin immunohistochemistry (IMH) method was used in this study. The characteristics of the antibodies used are the following: anti-IL-12 (ab106270, rabbit, working dilution 1:200, Abcam, Cambridge, UK); anti-IL-13 (sc-390676, mouse, working dilution 1:100, Santa Cruz Biotechnology Inc., Dallas, TX, USA); anti-IL-1β (ab2105, rabbit, working dilution 1:100, Abcam, Cambridge, UK); anti-SHH (ab53281, rabbit, working dilution 1:100, Abcam, Cambridge, UK); anti-NFkBp65 (orb37069, rabbit, working dilution 1:100, Biorbyt, Cambridge, UK); and anti-HSP60 (sc-1052, goat, working dilution 1:100, Santa Cruz Biotechnology Inc., Dallas, TX, USA).

A Leica DC 300F camera microscope was employed to examine the samples using bright-field microscopy. The slides were evaluated by two independent morphologists. Standard histological images were taken and analyzed with the Image-Pro Plus 7.0 image visualization software. The relative quantity of positive immunoreactive structures was assessed using a semi-quantitative grading method. In patient samples, a number of marker-positive cells were evaluated in the gallbladder epithelium, inflammatory infiltrate, and blood vessels (the wall and endothelium). In control samples, a number of marker-positive cells were evaluated in gallbladder epithelium and blood vessels (the wall and endothelium). Positively stained cells were counted by light microscopy and then graded using a scale containing the following values: 0—no positive structures (0%); 0/+—occasional positive structures (12.5%); +—few positive structures (25%); +/++—small to moderate number of positive structures (37.5%); ++—moderate number of positive structures (50%); ++/+++—moderate to numerous number of positive structures (62.5%); +++—numerous positive structures (75%); +++/++++—numerous to an abundance of positive structures (87.5%); and ++++—abundance of positive structures (100%) [48].

### 2.3. Statistics

Statistical analysis of the collected data was conducted using SPSS Statistics 29 (IBM, Burbank, CA, USA). The data were ranked as ordinal values, with 0 representing no positive structures observed in the visual field, 0.5 corresponding to occasional positive structures (0/+), and 1.0 indicating a few positive structures (+). The highest value, 4.0, corresponded to an abundance of positive structures (++++). Non-parametric tests were applied, and the Mann–Whitney U test was used to identify statistically significant differences in marker expression between the patient and control groups. Spearman’s rank-order correlation coefficient (Spearman’s rho, r_s_) was calculated to assess correlations between the different markers. Correlation strength was classified as very weak (r_s_ 0.00–0.30), weak (r_s_ 0.30–0.50), moderate (r_s_ 0.50–0.70), strong (r_s_ 0.70–0.90), or very strong (r_s_ 0.90–1.00). A strong positive correlation is associated with r_s_ value closer to +1, while a strong negative correlation is associated with r_s_ value closer to −1. In all analyses, a *p*-value of less than 0.05 was considered statistically significant.

## 3. Results

### 3.1. Routine Histology

**Figure 1:**
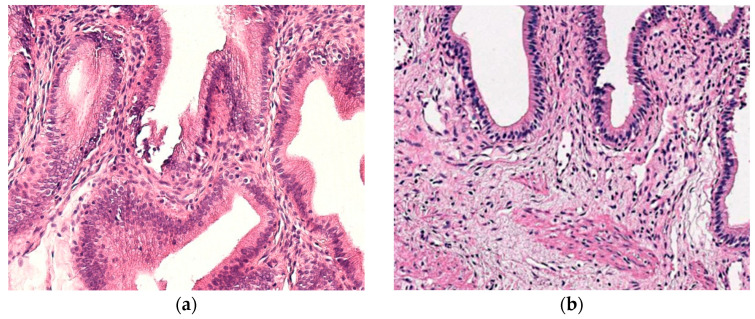
(**a**) Infiltrate of mononuclear cells and tissue oedema in the gallbladder wall of a patient sample. H&E, ×200. (**b**) No inflammatory changes are noted in the gallbladder wall of a control sample. H&E, ×200.

In all patient samples, mononuclear inflammatory cell infiltration was predominantly noted (Figure 1a). Other histological findings of patient tissues included oedema, muscular hypertrophy, and fibrotic changes throughout the gallbladder wall. In control samples, no inflammatory changes were seen (Figure 1b).

### 3.2. Immunohistochemistry

**Figure 2:**
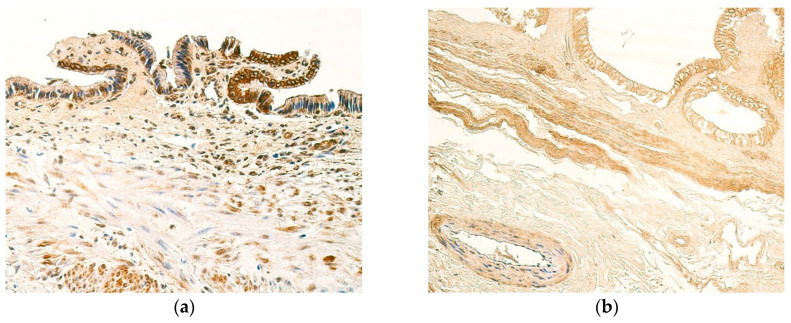
(**a**) Moderate number of IL-12-positive cells in the epithelium and subepithelial inflammatory infiltrate of a 17-year-old patient. IL-12 IMH, ×200. (**b**) Moderate to numerous weakly stained IL-12-positive cells in the epithelium and small to moderate numbers of IL-12-positive cells in the blood vessels of a control sample. IL-12 IMH, ×200.

IL-12 expression was consistently observed across patient samples, with a predominantly moderate number of IL-12-positive cells present in all tissue compartments—the epithelium, inflammatory infiltrate, and blood vessels (Figure 2a). In comparison, control samples showed small to moderate numbers of IL-12-positive cells in blood vessels, while the epithelium exhibited between a moderate number of and numerous IL-12-positive cells (Figure 2b), (Table 2 and Table 3).

**Figure 3:**
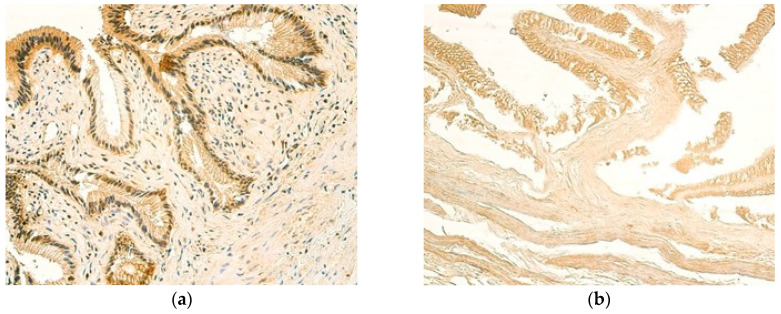
(**a**) A moderate number of IL-13-positive cells in the epithelium and few IL-13 positive cells in the inflammatory infiltrate of a 17-year-old patient. IL-13 IMH, ×200. (**b**) A moderate number of weakly stained IL-13-positive cells in the epithelium of a control sample. IL-13 IMH, ×200.

The epithelium of both patients and controls showed mostly moderate numbers of IL-13-positive cells, while blood vessels of patient samples revealed a higher expression of IL-13 than controls, with the modal value in patients being a moderate number of positive cells (Table 4 and Table 5) (Figure 3a,b). The number of IL-13-positive cells in inflammatory infiltrate varied, with few and numerous positive cells being observed most frequently (Table 4).

**Figure 4:**
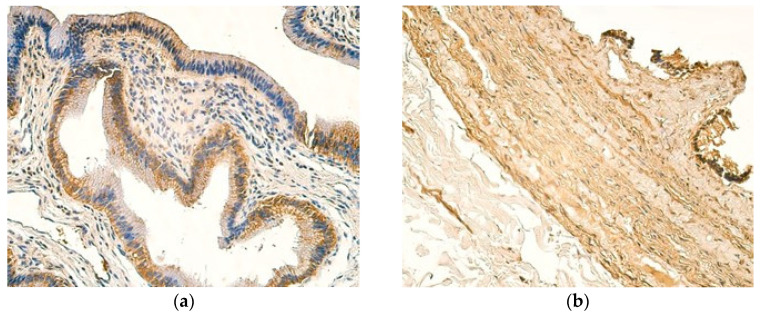
(**a**) Moderate number of IL-1β-positive cells in the epithelium and small to moderate numbers of IL-1β-positive cells in the inflammatory infiltrate of a 13-year-old patient. IL-1β IMH, ×200. (**b**) Numerous IL-1β -positive cells in the epithelium and a small to moderate number of IL-1β-positive cells in the blood vessels of a control sample. IL-1β IMH, ×200.

Interestingly, the expression of IL-1β was more prominent in the epithelium of control samples showing numerous IL-1β-positive cells, while the epithelium of patients expressed IL-1β at a moderate level (Figure 4a,b). Small to moderate numbers of blood vessel cells were stained positively for IL-1β in both patients and controls. The smallest amount of IL-1β -positive cells was noted in inflammatory infiltrate, where occasional and few IL-1β-positive cells were seen in most cases (Table 6 and Table 7).

**Figure 5:**
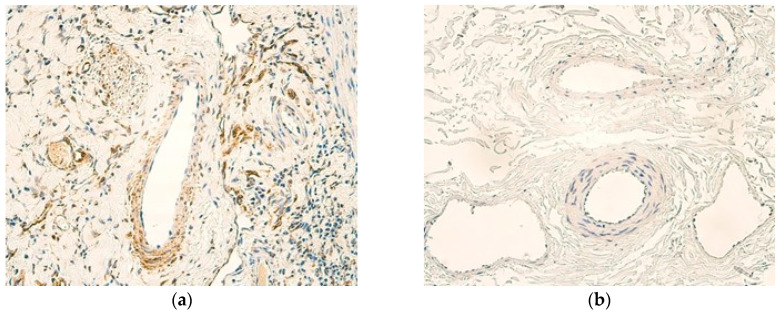
(**a**) Small to moderate number of SHH-positive cells in blood vessels and few SHH-positive cells in inflammatory infiltrate of a 12-year-old patient. SHH IMH, ×200. (**b**) No SHH-positive cells are seen in blood vessels of a control sample. SHH IMH, ×200.

Practically no SHH-positive cells could be seen in the gallbladders of control samples (Figure 5b), with only one sample showing occasional SHH-positive epitheliocytes (Table 8). The inflammatory infiltrate in patient samples was also frequently negatively stained for SHH. The gallbladder epithelium and blood vessels of patients, however, revealed SHH-positivity, where a moderate number of SHH-positive epitheliocytes could be noted, together with a small to moderate number of SHH-positive cells in the blood vessels seen in the majority of cases (Figure 5a), (Table 9).

**Figure 6:**
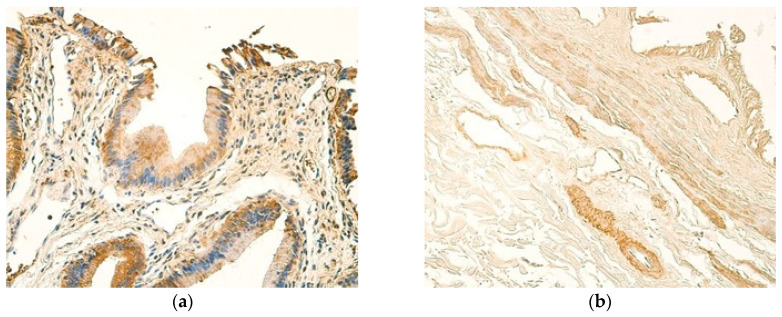
(**a**) Moderate number of NFkBp65-positive cells in the epithelium, blood vessels, and inflammatory infiltrate of a 13-year-old patient. NFkBp65 IMH, ×200. (**b**) Few weakly stained NFkBp65-positive cells are seen in the epithelium, with a moderate number of NFkBp65-positive cells in the blood vessels of a control sample. NFkBp65 IMH, ×200.

Patient samples exhibited mostly a moderate number of NFkBp65-positive cells in the epithelium, whereas control samples showed few positive gallbladder epitheliocytes. The blood vessel expression of NFkBp65 is relatively similar in both groups, where a moderate number of positive cells could be visualized (Figure 6a,b). The blood vessels of patients also frequently showed few NFkBp65-positive cells, meanwhile controls pertained to a small to moderate number of NFkBp65-positive cells (Table 10 and Table 11). From all tissue compartments examined in patient samples, inflammatory infiltrate showed the lowest expression of NFkBp65, with the modal value being a few NFkBp65-positive cells (Table 10).

**Figure 7:**
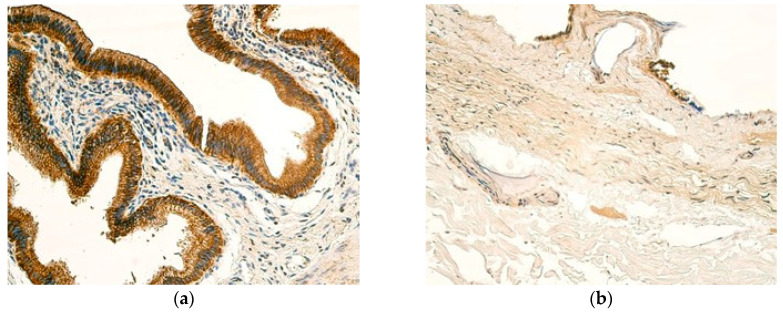
(**a**) A distribution of numerous numbers of to an abundance of HSP60-positive cells in the epithelium, a small to moderate number of HSP60-positive cells in blood vessels, and few HSP60-positive cells in the inflammatory infiltrate of a 13-year-old patient. HSP60 IMH, ×200. (**b**) Moderate to numerous HSP60-positive cells are seen in the epithelium, and few HSP60-positive cells are seen in the blood vessels of a control sample. HSP60 IMH, ×200.

The expression of HSP60 was the strongest in the epithelium of patients, where numerous positive cells were observed predominantly (Figure 7a). The epithelium of the controls also showed notable expression of HSP60, although it was not as strong as in patients, with moderate to numerous HSP60-positive cells observed frequently (Figure 7b). The blood vessels of patients were active producers of HSP60 as well, where all samples showed moderate expression of HSP60, or higher, with three samples containing numerous HSP60-positive cells. In contrast, a few cells of inflammatory infiltrate revealed HSP60 positivity, similar to the blood vessels of controls (Table 12 and Table 13).

An overview of the modal values of the marker expression in the gallbladder wall of patient and control samples is presented in Table 14.

### 3.3. Statistical Analysis

The Mann–Whitney U test showed statistically significant differences in the expression of the following markers between patients and controls: Il-12 in blood vessels (U = 8.5, *p* = 0.027); IL-1β in the epithelium (U = 37.0, *p* = 0.014); SHH in the epithelium (U = 3.5, *p* = 0.005) and in blood vessels (U = 2.5, *p* = 0.002); NFkBp65 in the epithelium (U = 0.0, *p* = 0.001); and HSP60 in blood vessels (U = 8.0, *p* = 0.027). It is worth noting that the expression of IL-1β was higher in the epithelium of controls than in the epithelium of patients (Table 15).

Multiple The italics has been removed strong and moderate positive correlations, as well as one very strong positive correlation between the studied markers, were found in patients. The strongest correlation was noted between the expression of IL-1β in inflammatory infiltrate and the expression of SHH in inflammatory infiltrate (r_s_ = 0.921, *p* < 0.001). In total, 6 strong positive correlations were detected between IL-13 and other markers—SHH, HSP60, IL-1β and NFkBp65; along with a total of four moderate positive correlations between IL-13 and SHH, IL-1β, and NFkBp65. Strong correlations were also seen between NFkBp65 and markers IL-12, IL-1β, and SHH. The remaining moderate correlations were found between IL-1β and markers IL-12 and SHH, as well as between different tissue compartments expressing IL-12 (Table 16).

## 4. Discussion

In this study, patient samples were found to express IL-12 moderately in all tissue compartments of the gallbladder wall, and blood vessels of patients showed a statistically significant increase in IL-12 expression when compared with controls. Although direct research on IL-12’s effects on gallbladder function is limited, its inflammatory role has implications for gallbladder diseases. Interestingly, Liu et al. [14] reported that higher circulating IL-12 levels are associated with gallstones in the adult population. It is well known that IL-12 mediates the TH1 immune response [49], and although TH1 cells are typically associated with the expression of IFN-γ, they also produce a different proinflammatory cytokine—tumor necrosis factor-alpha (TNF-α) [50]. It has been reported that TNF-α has a direct impact on the absorptive function of gallbladder epitheliocytes. Since proper absorption by the gallbladder partakes in the prevention of the formation of gallstones, disruptions in this function may contribute to the development of cholelithiasis and subsequent chronic cholecystitis [51]. Researchers also suggest that adaptive immunity, specifically the TH1-mediated immune response, is linked to the pathogenesis of cholesterol gallstone formation—in murine models, the downregulation of TH1 cytokines, chemokines, and receptors stopped the progression of cholesterol monohydrate crystals to cholesterol gallstones. Additionally, T-cell-derived inflammatory cytokines may participate in gallbladder wall muscle dysfunction [52]. Our study shows that the local inflammatory reaction in the gallbladder wall might be partially attributed to IL-12 actions, and the causative role of this cytokine in chronic pediatric calculous cholecystitis cannot yet be excluded.

In this study, there was no statistically significant difference detected in the expression of IL-13 between patients and controls; however, there were multiple strong positive correlations noted between IL-13 and other markers, namely SHH, HSP60, IL-1β, and NFkBp65, suggesting that IL-13 actions are involved in regulating the inflammatory processes responsible for chronic calculous cholecystitis. Despite typically being associated with TH2 immune responses and allergic inflammation, IL-13 exhibits important anti-inflammatory properties in various tissues as well [53,54]. Previous research demonstrates that IL-13 has the ability to inhibit a great number of pro-inflammatory cytokines, including IL-1β, IL-12, and TNF-α [55,56]. In addition, IL-13 interfered with NF-κB activation by preventing the nuclear translocation of the p65 subunit [56]. We suggest that in the gallbladder wall of patients with chronic cholecystitis, IL-13 might participate in feedback loops to regulate the intensity of chronic inflammation. Positive correlations with pro-inflammatory markers could represent compensatory mechanisms where IL-13 is upregulated in response to inflammation to mitigate excessive damage or promote resolution. It should be noted that chronic calculous cholecystitis involves long-standing inflammation and gallbladder wall remodeling [57]. IL-13 is a major cytokine responsible for promoting fibrotic processes and research indicates that IL-13 may directly promote the pro-fibrotic activities of macrophages and myofibroblasts [58]. This specific aspect of IL-13’s actions has been discussed in diseases such as systemic fibrosis, idiopathic pulmonary fibrosis, and others [59,60,61]. We suggest that IL-13’s well-known role in driving fibrosis might not require dramatic increases in expression but may act through interactions with other pathways to facilitate tissue remodeling and modulate gallbladder wall damage. Nevertheless, it has been reported that IL-13-producing cells can be present in healthy human lamina propria and IL-13Rα1, the cell surface receptor that binds IL-13, which is expressed constitutively in gut epithelium [62]. In our study, notable expression of IL-13 in the gallbladder wall of control samples might reveal the importance of anti-inflammatory effects in maintaining tissue homeostasis and uninterrupted gallbladder functions. Overall, IL-13 has been reported to exert dual roles—both protective and harmful—and it is still a challenge to confidently differ these actions in pathological conditions [62,63] including pediatric chronic calculous cholecystitis.

Interestingly, in this study, the expression of IL-1β was higher in the gallbladder epithelium of controls than in patients, and this difference was statistically significant. IL-1β contributes to the resolution of acute inflammation by promoting immune responses that clear the initiating trigger [64]. Higher levels of biliary IL-1β are found in patients with acute cholecystitis [65]. IL-1β is mainly produced by cells of the innate immune system [29], while in our study, inflammatory infiltrate showed predominantly occasional and few IL-1β-positive cells. These results suggest that local expression of this cytokine is not crucial for sustaining inflammation in chronic calculous cholecystitis. Possibly, the presence of gallstones and ongoing chronic inflammation might shift the local cytokine production toward other inflammatory mediators—in this research, an increase in IL-12, SHH, NFkBp65, and HSP60 was detected in the gallbladder wall of patients. The prominent expression of IL-1β in controls provokes the thought that IL-1β may serve a protective function in the gallbladder epithelium. It is known that IL-1β mediates the differentiation of T helper 17 (TH17) cells [28]. Research also reveals that TH17 cytokines play an important role in the gastrointestinal system—they are pivotal in maintaining intestinal homeostasis by providing defense against different pathogens and exhibiting protective functions during colonic mucosal injury [66]. Cholelithiasis has been associated with the presence of bacteria in bile, most commonly *Enterococcus* spp. and *E. coli* and *Klebsiella* spp. [67,68,69]; therefore, it could be possible that in healthy gallbladder walls, stronger expression of IL-1β is associated with a more efficient mucosal defense system. It should also be noted that a very strong positive correlation was detected between the expression of IL-1β and SHH in inflammatory infiltrate. The interaction of IL-1β and SHH can be viewed dually. Inflammasome formation is needed for activation of IL-1β, and this process can be induced by tissue damage [70]. It is possible that IL-1β produced in response to mechanical irritation and immune activation may stimulate SHH to promote epithelial regeneration and maintain the structural integrity of the gallbladder wall [33]. However, SHH also contributes to chronic inflammation by modulating immune responses [34], and this correlation might reflect a reciprocal relationship between the two factors with the aim of further sustaining inflammation.

It was seen in this study that expression of SHH is elevated in the epithelium and blood vessels of patients compared to controls. The difference in expression levels was also statistically significant in both tissue compartments. The increased expression of SHH in the gallbladder epithelium of patients may indicate its role in promoting epithelial repair and regeneration in response to chronic inflammation and mechanical irritation caused by gallstones. It has been previously reported that in adult organisms, the Hedgehog (HH) signaling is upregulated as a consequence of injury—this phenomenon is noted in multiple organ systems, including the respiratory and gastrointestinal systems [32]. SHH is known to support the homeostatic environment and facilitate the recovery of various tissues under stress conditions [71,72,73]. While the exact mechanism of SHH signaling involved in the regeneration process is still not fully understood, it is known that SHH acts by promoting crucial processes such as cell survival, proliferation, plasticity, or transdifferentiation [32]. In healthy controls, the absence of SHH-positive cells suggests that the pathway is not activated under normal physiological conditions, likely due to a lack of provoking stimuli. Additionally, the presence of SHH-positive cells in the blood vessels of patients may reflect its involvement in angiogenesis and vascular remodeling in the chronically inflamed gallbladder. SHH is known to promote the differentiation of endothelial cells through vascular endothelial growth factor (VEGF), and higher expression of SHH is associated with an increase in other vasculogenesis-related factors, such as fibroblast growth factor (FGF) and angiopoietins [74,75]. It could be possible that this model of SHH action is also applicable to gallbladder diseases, specifically chronic calculous cholecystitis. Although the actions of SHH described above are in accordance with an adaptive response of the gallbladder to chronic irritation and damage, overexpression of this signaling molecule is also connected to the aggravation of chronic inflammation, as seen in arthritis [76] and nonalcoholic steatohepatitis (NASH) [77]. Interestingly, in murine models, data suggest the opposite—application of SHH after central nervous system injury resulted in reduced immune cell migration and increased anti-inflammatory immune cell differentiation [78]. The anti-inflammatory effects of SHH are also supported by reduced HH signaling activity in the colonic mucosa of inflammatory bowel disease (IBD) patients [79]. Nonetheless, aberrant SHH signaling is associated with various tumors, including gallbladder carcinoma [80]; therefore, it is of great importance to maintain the local expression of SHH within the physiological intervals. Considering the possible dual effects of SHH, researchers discuss that actions of SHH can be described as tissue-dependent, and unknown environmental signals and cues from the surrounding tissue are determining whether SHH exerts an anti-inflammatory effect or promotes inflammatory responses [81]. Altogether, the distinct pattern of SHH expression in patient gallbladder tissues reflects the miscellaneous roles of SHH in inflammation, tissue remodeling, and vascular responses under chronic inflammation.

NF-kB is a critical transcription factor activated during inflammatory responses, and NFkBp65 is involved in the formation of the most common NF-kB heterodimer, therefore representing the canonical pathway [82]. Moderate expression of NFkBp65 in the epithelium of patient samples compared to relatively lower expression in controls likely reflects its activation in response to cellular stress and pro-inflammatory cytokines present in chronic calculus cholecystitis [83,84,85]. NF-kB signaling plays a dual role in cellular adaptation by coordinating responses to environmental changes and regulating immune function. Under physiological conditions, NF-kB facilitates metabolic reprogramming in immune cells to adapt to inflammatory signals. However, during prolonged inflammation, the hyperactivation of NF-kB drives excessive expression of inflammation-related genes [86], contributing to both the resolution and exacerbation of inflammatory processes. IL-1β is a known activator of the NF-kB pathway [87], and interestingly, NF-kB can, in turn, induce the production of IL-1β [40]. Among other target genes of NF-kB that are involved in immunomodulation is the IL-12 gene—signaling through the canonical pathway is associated with upregulated secretion of IL-12 [37,88]. Interactions between the NF-kB and Hedgehog pathways have also been described—SHH is a transcriptional target of NF-kB, and overactivation of this crosstalk is associated with a worse prognosis in some malignant diseases [89,90]. It has been researched that NF-kB signaling is crucial in organ injury related to cholestasis, and inhibition of NF-kB signaling and suppression of related pro-inflammatory cytokines had a protective effect [91]. The relatively low levels of NF-kBp65 in the inflammatory infiltrate of patient samples may suggest that NF-kB activation in these cells occurs transiently or is tightly regulated to prevent excessive immune activation. Alternatively, the inflammatory cells might rely on other signaling pathways. To highlight the complexity of NF-kB effects, it should be noted that the NF-kB pathway may also exert an anti-inflammatory role, incongruously, by limiting the production of pro-inflammatory cytokine IL-1β [92]. Interestingly, under normal conditions, inflammation is considered to be self-limiting; thus, the anti-inflammatory effects of NF-kB would participate in the restoration of the physiological state. However, dysfunction in these regulatory mechanisms may result in chronic inflammation and disease [93]. The results of our study are in accordance with previous research on crosstalk between the NF-kB pathway and other inflammatory factors and suggest that NF-kBp65 is significant in regulating morphopathogenetic processes in pediatric chronic calculous cholecystitis since a strong positive correlation was noted between the expression of NF-kBp65 and IL-1β in inflammatory infiltrate, as well as between NF-kBp65 and IL-12 in blood vessels and NF-kBp65 and SHH in inflammatory infiltrate of patients. Principally, the findings of this research suggest the composite role of NF-kBp65 in chronic calculous cholecystitis, highlighting its potential involvement in inflammatory signaling and its regulatory interactions with key tissue factors.

It was revealed that the strongest expression of HSP60 can be seen in the epithelium of patient samples; however, notable HSP60 presence was also detected in gallbladder epitheliocytes of the control group. The statistically significant differences in HSP60 expression in blood vessels between patients and controls further highlight its involvement in pathological processes associated with pediatric chronic calculous cholecystitis. HSP60 production is upregulated in response to cellular stress [94]. While it is traditionally considered to participate in correct protein folding and maintaining protein homeostasis [95], research suggests that HSP60 also serves as a messenger protein that leads to NF-kB-mediated survival gene expression in the nucleus [96]. Generally, HSP60 can be described as a pro-inflammatory factor in various diseases—this molecular chaperone is a ligand for toll-like receptors (TLRs) and one of its actions is to induce the activation of macrophages. As a consequence, inflammatory cytokines, such as IL-1β and IL-6, are produced via activation of the NF-kB pathway [44,97]. Relatively stronger expression of HSP60 in the gallbladder epithelium of chronic cholecystitis patients likely reflects its role in protecting epithelial cells from chronic stress induced by gallstones, while also potentially contributing to sustaining the inflammation in the gallbladder wall. Additionally, a strong positive correlation was detected between the expression of HSP60 and IL-13 in gallbladder wall of patients. Along with its proinflammatory properties, HSP60 can also have immunoregulatory effects—HSP60 suppresses T-cell migration and modulates cytokine secretion to favor a TH2-dominant immune response, thus contributing to the determination of the inflammatory process [98]. It is possible that the correlation between HSP60 and IL-13 represents the switch to TH2 cytokine production in the gallbladder wall. Vascular expression of HSP60 has been described in cardiovascular diseases—it is translocated to the cell surface and acts as a danger signaling molecule [99]. HSP60 is known to induce the production of cyclooxygenase-2 (COX-2) and nitric oxide synthase (NOS-2) in endothelial cells, therefore amplifying the inflammation [100]. In the present study, the significant upregulation of HSP60 in blood vessels of the gallbladder wall suggests its involvement in endothelial activation in cases of chronic calculous cholecystitis. The relatively low HSP60 expression in the inflammatory infiltrate of both patients and controls may indicate that HSP60’s primary role is within structural tissue compartments (epithelium and vasculature) rather than infiltrating immune cells. In controls, the presence of HSP60 might represent its function in maintaining basal cellular homeostasis under physiological conditions.

All things considered, the gallbladder wall epithelium and blood vessels of patients with chronic calculous cholecystitis expressed all of the examined immunomodulatory tissue factors, namely, IL-12, IL-13, IL-1β, SHH, NFkBp65, and HSP60. Inflammatory infiltrate of patients, however, revealed more active production of IL-12 and IL-13. These findings show that inflammatory response and regulation are sustained in the barrier surface, as well as in deeper layers of the gallbladder wall in cases of pediatric chronic calculous cholecystitis.

This study has a few limitations, the main one being a relatively small number of patient and control samples. A larger sample size could lead to more precise results and could further emphasize the differences between patients and controls; however, obtaining healthy gallbladder tissues from pediatric patients raises ethical concerns. In this research, only immunohistochemical examination was conducted, although other methods, such as ELISA or detection of certain gene expression by PCR, could assist in a more detailed and quantitative description of immunomodulatory tissue factors in chronic calculous cholecystitis. While the study identifies differences in tissue factor expression, it does not explore the functional impact of these molecules on immune or cellular responses. Functional assays could help establish the specific roles of these factors in disease pathogenesis. It would also be valuable to compare the local expression of studied tissue factors to serum concentrations and observe potential patterns that could participate in the sustained inflammation. Also, this study focuses on a single time point, limiting the understanding of how the expression of immunomodulatory factors evolves throughout the progression of pediatric chronic calculous cholecystitis. A longitudinal approach could offer insights into the temporal dynamics of these factors during the disease course. It should be mentioned that the study population is restricted to pediatric patients, and the results may not be generalizable to adult cases of chronic calculous cholecystitis or to diverse populations with varying genetic or environmental backgrounds.

## 5. Conclusions

The increased number of NFkBp65, IL-12, and HSP60 positive cells in patient gallbladder tissue suggests a significant role of these tissue factors in driving immune modulation and sustaining the inflammation in pediatric chronic calculous cholecystitis.

The notable expression of SHH in patient gallbladder tissue indicates its part in tissue regeneration and repair processes, as well as in modulating inflammation and vascular responses in calculous cholecystitis.

The significant positive correlations between the factors studied highlight the im-portance of their coordinated interaction and intricate crosstalk in the morphopathogenesis of calculous cholecystitis.

## Figures and Tables

**Table 1:** Overview of the main properties of the markers studied in this research.

Marker	Main Properties
IL-12	Induces differentiation of TH1 cells, leading to production of IFN-γ Promotes cellular immunity
IL-13	Product of TH2 cells Antagonizes TH1 mediated inflammation Role in eosinophil trafficking and fibrosis
IL-1β	Major proinflammatory cytokine Role in TH17 cell differentiation
SHH	Promotes tissue regeneration and repair Role in regulation of immune processes by affecting immune cell signaling and differentiation
NFkBp65	Controls main cellular processes in immune cells and induces production of inflammatory cytokines
HSP60	Maintains correct protein folding Extracellular forms promote cytokine production and TLR expression

Abbreviations: IL-12—interleukin-12; IL-13—interleukin-13 (IL-13); IL-1β—interleukin-1β; SHH—sonic hedgehog protein; NFkBp65—nuclear factor NF-kappa-B p65 subunit; HSP60—heat shock protein 60; TH1—T helper 1; IFN-γ—interferon gamma; TH2—T helper 2; TH17—T helper 17; TLR—toll like receptors.

**Table 2:** Expression of IL-12 in the gallbladder epithelium, inflammatory infiltrate, and blood vessels of patient samples.

Number of Specimen	Epithelium	Inflammatory Infiltrate	Blood Vessels
1	++	+/++	++/+++
2	++	0/+	+
3	+/++	+	+/++
4	++	++	++
5	+++	++	++
6	++	++/+++	+++
7	+/++	++	+++
8	+++	+++	+++
9	++	++	++
10	+++	+	++/+++
11	−	0/+	++
Modal value	++	++	++

Abbreviations: 0/+—occasional positive structures (12.5%); +—few positive structures (25%); +/++—small to moderate number of positive structures (37.5%); ++—moderate number of positive structures (50%); ++/+++—moderate to numerous number of positive structures (62.5%); +++—numerous positive structures (75%); IL-12—interleukin-12.

**Table 3:** Expression of IL-12 in the gallbladder epithelium and blood vessels of control samples.

Number of Specimen	Epithelium	Blood Vessels
1	++	0/+
2	++/+++	+/++
3	−	+/++
4	+++	++
5	++/+++	+/++
Modal value	++/+++	+/++

Abbreviations: 0/+—occasional positive structures (12.5%); +/++—small to moderate number of positive structures (37.5%); ++—moderate number of positive structures (50%); ++/+++—moderate to numerous number of positive structures (62.5%); IL-12—interleukin-12.

**Table 4:** Expression of IL-13 in the gallbladder epithelium, inflammatory infiltrate, and blood vessels of patient samples.

Number of Specimen	Epithelium	Inflammatory Infiltrate	Blood Vessels
1	++	++	0/+
2	+++	+++	++
3	++	+/++	++
4	++	0/+	++
5	+++	0/+	+
6	++	++	+/++
7	++/+++	+++	++/+++
8	+++/++++	+++	++/+++
9	+	+	++
10	++	+	++
11	−	+	++
Modal value	++	+; +++	++

Abbreviations: 0/+—occasional positive structures (12.5%); +—few positive structures (25%); +/++—small to moderate number of positive structures (37.5%); ++—moderate number of positive structures (50%); ++/+++—moderate to numerous number of positive structures (62.5%); +++—numerous positive structures (75%); IL-13—interleukin-13.

**Table 5:** Expression of IL-13 in the gallbladder epithelium and blood vessels of control samples.

Number of Specimen	Epithelium	Blood Vessels
1	+	+
2	+/++	+/++
3	++	++
4	++	+/++
5	++	+/++
Modal value	++	+/++

Abbreviations: +—few positive structures (25%); +/++—small to moderate number of positive structures (37.5%); ++—moderate number of positive structures (50%); IL-13—interleukin-13.

**Table 6:** Expression of IL-1β in the gallbladder epithelium, inflammatory infiltrate, and blood vessels of patient samples.

Number of Specimen	Epithelium	Inflammatory Infiltrate	Blood Vessels
1	++	+/++	+/++
2	++	+++	++
3	+/++	+	+/++
4	++	0/+	+/++
5	+++	+	++
6	0/+	++/+++	++/+++
7	++	+++	++
8	++	++/+++	++/+++
9	++	0/+	+
10	++/+++	0/+	++
11	−	+	+/++
Modal value	++	0/+; +	+/++; ++

Abbreviations: 0/+—occasional positive structures (12.5%); +—few positive structures (25%); +/++—small to moderate number of positive structures (37.5%); ++—moderate number of positive structures (50%); ++/+++—moderate to numerous number of positive structures (62.5%); +++—numerous positive structures (75%); IL-1β—interleukin-1beta.

**Table 7:** Expression of IL-1β in the gallbladder epithelium and blood vessels of control samples.

Number of Specimen	Epithelium	Blood Vessels
1	++/+++	+/++
2	+++	++
3	−	+/++
4	+++	+/++
5	+++	+/++
Modal value	+++	+/++

Abbreviations: +/++—small to moderate number of positive structures (37.5%); ++—moderate number of positive structures (50%); ++/+++—moderate to numerous number of positive structures (62.5%); +++—numerous positive structures (75%); IL-1β—interleukin-1beta.

**Table 8:** Expression of SHH in the gallbladder epithelium and blood vessels of control samples.

Number of Specimen	Epithelium	Blood Vessels
1	0	0
2	0	0
3	0	0
4	0	0
5	0/+	0
Modal value	0	0

Abbreviations: 0—no positive structures (0%); 0/+—occasional positive structures (12.5%); SHH—sonic hedgehog protein.

**Table 9:** Expression of SHH in the gallbladder epithelium, inflammatory infiltrate, and blood vessels of patient samples.

Number of Specimen	Epithelium	Inflammatory Infiltrate	Blood Vessels
1	++	0/+	0/+
2	++	++/+++	+/++
3	+/++	0/+	0/+
4	++	0	+
5	+++	0/+	0
6	+	+	+
7	+	+	+/++
8	++	++	++
9	0	0	+
10	++/+++	0	++
11	−	0	+/++
Modal value	++	0	+; +/++

Abbreviations: 0—no positive structures (0%); 0/+—occasional positive structures (12.5%); +—few positive structures (25%); +/++—small to moderate number of positive structures (37.5%); ++—moderate number of positive structures (50%); ++/+++—moderate to numerous number of positive structures (62.5%); +++—numerous positive structures (75%); SHH—sonic hedgehog protein.

**Table 10:** Expression of NFkBp65 in the gallbladder epithelium, inflammatory infiltrate, and blood vessels of patient samples.

Number of Specimen	Epithelium	Inflammatory Infiltrate	Blood Vessels
1	++	++	++
2	+/++	++	+
3	++	+	++
4	+++	+	+
5	+++	+	+
6	++	+/++	++
7	+/++	++	+++
8	++	++	+++
9	++	+	+
10	++	0/+	++
11	++	+	+/++
Modal value	++	+	+; ++

Abbreviations: 0/+—occasional positive structures (12.5%); +—few positive structures (25%); +/++—small to moderate number of positive structures (37.5%); ++—moderate number of positive structures (50%); +++—numerous positive structures (75%); NFkBp65—nuclear factor NF-kappa-B p65 subunit.

**Table 11:** Expression of NFkBp65 in the gallbladder epithelium and blood vessels of control samples.

Number of Specimen	Epithelium	Blood Vessels
1	+	+/++
2	+	++
3	−	+/++
4	+	++
5	+	+
Modal value	+	+/++;++

Abbreviations: +—few positive structures (25%); +/++—small to moderate number of positive structures (37.5%); ++—moderate number of positive structures (50%); NFkBp65—nuclear factor NF-kappa-B p65 subunit.

**Table 12:** Expression of HSP60 in the gallbladder epithelium, inflammatory infiltrate, and blood vessels of patient samples.

Number of Specimen	Epithelium	Inflammatory Infiltrate	Blood Vessels
1	+++/++++	+	+/++
2	+++	+/++	++
3	+++	+	++
4	+++	0/+	+++
5	+++	+	++/+++
6	++	0/+	++
7	+	+	++
8	++/+++	++	+++
9	++	0/+	+/++
10	+++/++++	+	+++
11	−	+	++/+++
Modal value	+++	+	++

Abbreviations: 0/+—occasional positive structures (12.5%); +—few positive structures (25%); +/++—small to moderate number of positive structures (37.5%); ++—moderate number of positive structures (50%); ++/+++—moderate to numerous number of positive structures (62.5%); +++—numerous positive structures (75%); +++/++++—numerous to abundance of positive structures (87.5); HSP60—heat shock protein 60.

**Table 13:** Expression of HSP60 in the gallbladder epithelium and blood vessels of control samples.

Number of Specimen	Epithelium	Blood Vessels
1	+++	+
2	++/+++	+
3	−	+
4	++	++
5	++/+++	++
Modal value	++/+++	+

Abbreviations: +—few positive structures (25%); ++—moderate number of positive structures (50%); ++/+++—moderate to numerous number of positive structures (62.5%); +++—numerous positive structures (75%); HSP60—heat shock protein 60.

**Table 14:** Summary of modal values of marker expression in the gallbladder wall of patient and control samples.

Marker	Epithelium	Blood Vessels	Inflammatory Infiltrate
Patient Samples			
IL-12	++	++	++
IL-13	++	++	+; +++
IL-1β	++	+/++; ++	0/+; +
SHH	++	+; +/++	0
NFkBp65	++	+; ++	+
HSP60	+++	++	+
Control Samples			
IL-12	++/+++	+/++	−
IL-13	++	+/++	−
IL-1β	+++	+/++	−
SHH	0	0	−
NFkBp65	+	+/++; ++	−
HSP60	++/+++	+	−

Abbreviations: 0—no positive structures (0%); 0/+—occasional positive structures (12.5%); +—few positive structures (25%); +/++—small to moderate number of positive structures (37.5%); ++—moderate number of positive structures (50%); ++/+++—moderate to numerous number of positive structures (62.5%); +++—numerous positive structures (75%); IL-12—interleukin-12; IL-13—interleukin 13; IL-1β—interleukin-1 beta; SHH—sonic hedgehog protein; NFkBp65—nuclear factor NF-kappa-B p65 subunit; HSP60—heat shock protein 60.

**Table 15:** Significant differences in marker expression between patient and control samples.

Marker and Tissue Compartment	U Value	*p*-Value
IL-12 (blood vessels)	8.5	0.027
IL-1β (epithelium)	37.0	0.014
SHH (epithelium)	3.5	0.005
SHH (blood vessels)	2.5	0.002
NFkBp65 (epithelium)	0.0	0.001
HSP60 (blood vessels)	8.0	0.027

Abbreviations: U—Mann–Whitney U test; *p*-value—*p*-values < 0.05 are considered statistically significant; IL-12—interleukin-12; IL-1β—interleukin-1 beta; SHH—sonic hedgehog protein; NFkBp65—nuclear factor NF-kappa-B p65 subunit; HSP60—heat shock protein 60.

**Table 16:** Summary of Spearman’s rank-order correlation analysis to determine the moderate (r_s_ = 0.5–0.7), strong (r_s_ = 0.7–0.9), and very strong (r_s_ = 0.9–1.0) positive relationship between the number of marker-containing cells in different tissue compartments in patient samples.

Compared Markers and Tissue Compartments		*n*	r_s_	*p*-Value
Very strong correlation (r_s_ = 0.9–1.0)				
IL-1β (Inflammatory Infiltrate)	SHH (Inflammatory Infiltrate)	11	0.921	<0.001
Strong correlation (r_s_ = 0.7–0.9)				
IL-12 (Blood Vessels)	NFkBp65 (Blood Vessels)	11	0.745	0.008
IL-13 (Epithelium)	SHH (Inflammatory Infiltrate)	10	0.741	0.014
IL-13 (Epithelium)	HSP60 (Inflammatory Infiltrate)	10	0.800	0.005
IL-13 (Inflammatory Infiltrate)	IL-1β (Inflammatory Infiltrate)	11	0.874	<0.001
IL-13 (Inflammatory Infiltrate)	SHH (Inflammatory Infiltrate)	11	0.834	0.001
IL-13 (Inflammatory Infiltrate)	NFkBp65 (Inflammatory Infiltrate)	11	0.849	<0.001
IL-13 (Blood Vessels)	SHH (Blood Vessels)	11	0.725	0.012
IL-1β (Inflammatory Infiltrate)	NFkBp65 (Inflammatory Infiltrate)	11	0.884	<0.001
SHH (Inflammatory Infiltrate)	NFkBp65 (Inflammatory Infiltrate)	11	0.823	0.002
Moderate correlation (r_s_ = 0.5-0.7)				
IL-12 (Epithelium)	IL-1β (Epithelium)	10	0.656	0.039
IL-12 (Inflammatory Infiltrate)	IL-12 (Blood Vessels)	11	0.671	0.024
IL-13 (Epithelium)	IL-1β (Inflammatory Infiltrate)	10	0.636	0.048
IL-13 (Epithelium)	IL-1β (Blood Vessels)	10	0.674	0.033
IL-13 (Epithelium)	SHH (Epithelium)	10	0.655	0.040
IL-13 (Inflammatory Infiltrate)	NFkBp65 (Blood Vessels)	11	0.626	0.039
IL-1β (Blood Vessels)	SHH (Inflammatory Infiltrate)	11	0.696	0.017

Abbreviations: *n*—pairs analyzed in Spearman’s rank-order correlation analysis; r_s_—correlation coefficient (Spearman’s rho); *p*-value—*p*-values < 0.05 are considered statistically significant; IL-12—interleukin-12; IL-13—interleukin-13; IL-1β—interleukin-1 beta; SHH—sonic hedgehog protein; NFkBp65—nuclear factor NF-kappa-B p65 subunit; HSP60—heat shock protein 60.

## Data Availability

The data presented in this study are available upon request from the corresponding author.
